# A Sustainable Lifestyle Intervention Among Office Workers: Cluster Randomized Pilot and Feasibility Study

**DOI:** 10.2196/82061

**Published:** 2026-05-07

**Authors:** Oskar Halling Ullberg, Annika Tillander, Katarina Bälter

**Affiliations:** 1 Department of Health Science, Innovation and Design, Mälardalen University, Universitetsplan 1, Västerås, 722 20, Sweden, 46 79-0660901; 2 Department of Statistics and Machine Learning, Linköping University, Linköping, Sweden; 3 Department of Medical Epidemiology and Biostatistics, Karolinska Institute, Stockholm, Sweden

**Keywords:** workplace, office, diet, carbon dioxide equivalents, behavior change, physical activity

## Abstract

**Background:**

Society faces multiple challenges, including lifestyle diseases and global climate change. Framing health education within sustainable development may enhance motivation for behavior change because proenvironmental behaviors, as well as healthy behaviors, often rely on the same behavior change principles. Combining these perspectives may therefore reinforce health behaviors and climate-friendly choices.

**Objective:**

This pilot study aims to explore changes in dietary intake, diet-related carbon footprint, and physical activity among office workers receiving sustainable plus healthy lifestyle (sustainable lifestyle arm) or healthy lifestyle education (healthy lifestyle arm) alone. It also aims to assess the feasibility of the intervention functions, including workshop attendance rate, participants’ dietary goals, social support, and facilitators and barriers to behavior change.

**Methods:**

A 2-armed participant-blinded cluster randomized study, including an experimental intervention arm (sustainable lifestyle; n=19) and a control intervention arm (healthy lifestyle; n=14), was conducted in Sweden. The study lasted 8 weeks and included 6 workplace-based workshops and was framed by the behavioral change wheel and the socioecological model. Diet, carbon footprint, and physical activity were assessed using the web-based questionnaires Meal-Q and Active-Q. Attendance rate, individual goals, social support, and facilitators and barriers were assessed using printed questionnaires.

**Results:**

The reduction of total diet-related carbon dioxide equivalents (CO_2_e) was 0.8 kg and 0.4 kg per day for the sustainable and healthy lifestyle arm, respectively. Also, there was a statistically significant interaction between time and lifestyle when the carbon footprint was expressed as a qualitative aspect of diet, that is, CO_2_e kg per 1000 kcal per day (*P*=.05). Moreover, the intake of vitamin C, a marker for fruits and vegetables, increased to 8.0 and 12.5 mg per 1000 kcal per day for the sustainable and healthy lifestyle arms, respectively. In addition, total sedentary time decreased by 0.4 hours per day in the sustainable lifestyle arm, but not in the healthy lifestyle arm. This indicates that the educational workshops in respective arms had different impacts on health behavior over time. Minor differences were found in dietary goals, with the sustainable lifestyle arm setting more goals related to ecological and vegetarian foods. No differences were seen between arms regarding barriers or facilitators.

**Conclusions:**

This study suggests that embedding healthy lifestyle recommendations within a sustainable development context may be an efficient way to reduce carbon footprint and increase healthy behavior among office workers. Given the ongoing global epidemic of metabolic diseases, climate change, and environmental degradation, promoting a sustainable lifestyle in a workplace context has the potential to counteract these trends.

## Introduction

Unhealthy behaviors, including poor food habits and insufficient physical activity, increase the risk of metabolic diseases and contribute to a substantial economic burden on the health care system [1,2], as well as for corporations through increased employee absenteeism and reduced productivity [3,4]. Moreover, a nonsustainable western lifestyle relying on the excessive production of food and goods, passive transportation, and the heating and cooling of buildings, among other things, contributes substantially to increased carbon footprints through greenhouse gas emissions, deforestation, and increased land use, as well as pollution, traffic noise, and environmental degradation [5,6].

Epidemiological studies consistently show that high consumption of plant-based foods such as vegetables, fruits, legumes, and whole-grain products and physical activity reduces the incidence of noncommunicable diseases like obesity, cardiovascular diseases, type 2 diabetes, and certain types of cancer [7-11]. Moreover, reviews at the time of this writing demonstrate the importance of active transportation to decrease traffic congestion, greenhouse gas emissions, air pollution, and noise [12-14]. Thus, relatively minor behavior changes among a large proportion of the population, such as a reduction in beef consumption, along with increased physical activity, including active commuting, would drastically impact public health and yield substantial environmental benefits [10,13,15,16].

Given that more than 50% of the 5.1 million employees working in Sweden are office workers, also described as white-collar workers [17,18], the office is a strategic arena for health promotion. Previous workplace dietary interventions in the form of counseling, education, and on-site group activities have shown substantial changes in employees’ food habits and health-related outcomes [19-21]. Workplace interventions targeting reduced sedentary behavior and increased physical activity at work [22-24], weight loss, and improvement of metabolic syndrome markers have also shown promising results [25,26], regardless of workplace type. These interventions resulted in improved employee health and financial return for the employer, especially reduced absenteeism [3,27,28]. Although workplace interventions have targeted healthier behaviors, they rarely combine established health behavior change frameworks with approaches that encourage proenvironmental behaviors, such as choosing foods with a low carbon footprint or increasing active transportation. Integrating these 2 perspectives remains uncommon, even though both influence diet and physical activity behaviors [29]. This gap limits our understanding of how workplace interventions can support behaviors that benefit both individual health and environmental sustainability. Thus, there is a need for well-designed, theory-informed workplace interventions specifically targeting a healthy and sustainable lifestyle among office workers [19,22,30,31].

The built environment, building design, social, and organizational factors within the office workplace, along with individual capability and motivation, are crucial for behavioral change [31,32]. Moreover, interventions using physical prompts for stair climbing or maps marked with walking routes, are more effective than interventions solely focusing on information dissemination and individual behaviors [33]. Here, we report from the pilot phase of the Sustainable Office Intervention (SOFIA) [34]. The intervention hypothesizes that office workers who receive healthy lifestyle education framed within the context of sustainable development will exhibit greater motivation for changes in dietary and physical activity behaviors compared to those who receive education focused solely on healthy behaviors. Specifically, we aim to assess changes in (1) diet-related carbon footprint, expressed as CO_2_e (diet-related carbon dioxide equivalents) and a marker of sustainable food habits; (2) intake of energy and nutrients; and (3) physical activity and inactivity, including the use of active commuting. Second, we aim to evaluate the feasibility of intervention functions in the form of participants’ workshop attendance and perceived social support, as well as differences in goals, facilitators, and barriers for behavior change.

## Methods

### Study Design

This pilot study compares a healthy plus sustainable lifestyle intervention arm with a healthy lifestyle control intervention arm, hereafter referred to as the sustainable lifestyle (SL) and healthy lifestyle (HL) arm, respectively. After randomization, each arm received an explanation of the respective lifestyle concepts during workshop number 1. Participants in the HL arm were introduced to national recommendations that emphasize eating a varied diet rich in whole grains, fruits, and vegetables, along with limited consumption of red and processed meat, and engaging in 150-300 minutes of moderate-to-vigorous physical activity each week. Participants in the SL arm received the same health recommendations, framed within information on behaviors that also reduce environmental impact. This included choosing foods with lower greenhouse gas emissions, considering organically produced foods, and understanding the environmental benefits of active commuting such as walking or cycling. Because a pilot study may be considered a subset of feasibility studies, this study evaluates both the piloted trial procedures (eg, primary outcomes, delivery of workshops at the office locations, and intervention functions) and the feasibility aspects (eg, recruitment data, participant workshop attendance rate, and alignment of goals set by participants) to inform the design of a future full-scale SOFIA randomized controlled trial. A detailed study protocol of the SOFIA study as a full-scale cluster randomized, single-blind controlled trial has been described previously [34], and data have been reported according to the CONSORT (Consolidated Standards of Reporting Trials) checklist (Checklist 1).

### Setting

This study was co-created with 2 offices in Sweden and part of a large body of research within the Concepts for the Sustainable Office of the Future (SOFCO) project. Office A was located in a medium-sized city and worked in real estate development. At the time of the investigation, the office included approximately 35 office workers. The workspace consisted of modern coworking areas, shared workspaces, meeting rooms, and social areas. Office B was located in a large city and delivered facility management and property management services. At the time of the intervention, the office contained approximately 400 office workers. Although larger, Office B featured workspaces similar to those in Office A, including modern offices designed for flexible work and remote work opportunities, in line with the new ways of working, that is, employees are free to work from anywhere and anytime, and the office lacks individual and assigned office space for each person [35]. Both offices were situated in urban environments on 2 or more floors in their respective buildings, surrounded by restaurants, shopping centers, and fitness facilities and within walking distance to public transport and parks. For additional details about the setting, see Ullberg et al [36].

### Recruitment

Participants were recruited through a systematic multistep procedure. First, study information was communicated to managers at each office, who then shared the information internally. Second, recruitment advertisements were posted at the offices. Third, all office employees received an email invitation to participate. Finally, members of the research team visited the workplaces in person to provide information and recruit participants on site. Employees who expressed interest completed a short web-based screening questionnaire provided through the advertisement. Eligibility criteria included being at least 18 years of age, having access to a smartphone, and working 20 hours or more per week in one of the 2 offices. Upon confirmation of interest, participants were invited by email to attend a baseline assessment meeting at their workplace one week before the first educational workshop. The aim of the meeting was to provide information about the study, sign the informed consent form, and do baseline assessments.

### Randomization and Sample Size

After the baseline assessment, the 2 office sites were randomized as clusters using a computer-generated random number generator in the statistical software R (R Core Team). This allowed all participants to be assigned to either the sustainable or healthy arm based on their office’s geographical location [34]. Data collection was conducted in the fall of 2022 and repeated for a second cohort in the spring of 2023. Because the study included only 4 small clusters in total, the process functioned as 2 separate 2-arm allocations, each with equal probability of assignment. Note that the offices (A and B) received both interventions (SL and HL), but at different time points, as the randomization was repeated for each cohort. Participants were not aware of their allocation, as the different office-based clusters were geographically separated. However, the research team was not blinded and had access to files identifying each study arm allocation status, as they had to coordinate the intervention and data collection. We used purposive sampling, as only employees from the 2 companies with established agreements were eligible and invited to participate in the study. However, key outcome measures from this pilot study will be used to inform future power calculations and determine the required sample size of the future upscaled study, as outlined in the study protocol [34]. In total, 37 participants were recruited, of which 21 were included in the SL arm and 16 in the HL arm. The uneven distribution of participants in the SL and HL arms, respectively, was a result of the cluster randomization process, since different numbers of people signed up at the different offices. Given the small sample size and unequal group allocation, the study is not powered to detect between-group differences; therefore, all results should be regarded as exploratory.

### Education Intervention and Workshops

**Figure 1.**
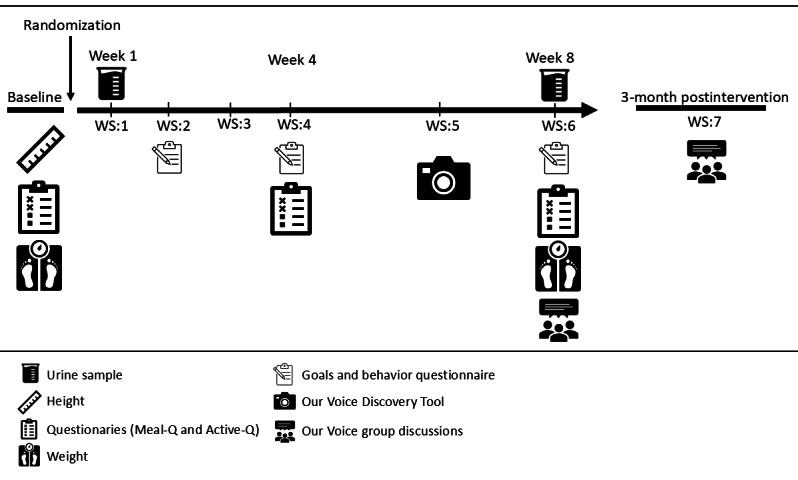
Timeline for the 8-week intervention study Sustainable Office Intervention (SOFIA) with 6 educational workshops (WS) and a follow-up meeting 2‐3 months after the intervention ended. WS: workshop.

The intervention lasted for 8 weeks and consisted of 6 interactive educational workshops that occurred every week for the first 4 weeks and then every second week for the last 4 weeks, facilitated by study personnel (OHU and KB). Diet and physical activity data were collected at baseline and just before workshops 4 and 6 to make sure that data were assessed according to the timeline shown in Figure 1, and if needed, participants were reminded at the workshop. Data addressing the secondary aim—namely, evaluating the feasibility of the intervention functions—were collected at workshops 2, 4, and 6 as part of the behavioral change support activities. Additionally, participants in both study arms used the Stanford Healthy Neighborhood Discovery Tool (Discovery Tool) [37] to address the social-ecological aspects of behavior change; these data were collected between workshops 5 and 6. However, the results have been presented elsewhere [36]. Moreover, urine samples were collected for analysis of pesticide residues, as an indicator of consumption of organic foods, at baseline and workshop 6, but the result is beyond the scope of this paper.

### A Framework for Behavior Change

Michie et al [32,38] Behavioral Change Wheel and techniques from the behavior change taxonomy served as the framework for this intervention, based on the COM-B (capability, opportunity, and motivation to support behavior change) model, which explains individual behavior change through 3 fundamental mechanisms: capability (C), opportunity (O), and motivation (M), which together support behavior change (B). The interactive educational workshops included discussions about how to change behavior at the individual level to achieve a more sustainable and healthier lifestyle, respectively. In the context of this study, capability (C) referred to knowledge, planning, and culinary skills related to a healthy and sustainable lifestyle; opportunity (O) referred to food options at work and areas for engaging in physical activity; motivation (M) referred to personal factors that motivate behavioral change (B), such as a desire to improve one’s health, increase work productivity, reduce one’s global carbon footprint, and contribute to reducing local food waste. In brief, workshops 1-6 used COM-B-based intervention functions to support behavior change. Education was delivered through framing and reframing, highlighting that sustainable lifestyle choices can also promote health in the SL arm. Environmental restructuring was introduced by using the workplace as a social arena and through the Our Voice Discovery tool (Stanford University); moreover, by serving lunch during the workshops to model recommended eating practices aligned with the nutritional recommendations of the Nordic Nutrition Recommendations (NNR) [39]. Workshops 2 and 4 emphasized incentivization through goal setting and used prompts and cues, including guidance on interpreting food labels such as the Swedish Keyhole symbol and KRAV (the Swedish label for organically produced foods). Throughout workshops 1-7, enablement and action planning were supported by education and the digital behavioral package (guidelines, food labels, and recipes to support adherence to the recommended advice about sustainable and healthy lifestyles, respectively). For educational content, see Table 1, and for an even more detailed description of program theory, supporting factors, and intervention functions, see published study protocol [34].

**Table 1.** Educational content of the Sustainable Office Intervention (SOFIA) pilot workshops by intervention arm.

Workshop	Sustainable lifestyle	Healthy lifestyle
1.	Introduction to a sustainable lifestyle. Global food consumption and disease burden. The impact of food production and consumption on climate and environmental issues. Dietary guidelines with focus on recommended intake of red meat and processed meat as well as on fruits and vegetables, and fiber. The recommended proportion of energy from different macronutrients (fat, protein, and carbohydrates). The role of energy balance for maintaining a normal body weight. Win-win situation between climate and healthy aspects when eating plant-based food.	Introduction to a healthy lifestyle. Global food consumption and disease burden. Dietary guidelines with focus on recommended intake of red meat and processed meat as well as on fruits and vegetables, and fiber. The recommended proportion of energy from different macronutrients (fat, protein, and carbohydrates). The role of energy balance for maintaining a normal body weight.
2.	The climate and environmental impact of organic farming versus conventional farming. Consumption of organic products. How organic products are labeled on a national and European level.	The recommended proportion of energy from different macronutrients (fat, protein, and carbohydrates). The role of energy balance for maintaining a normal body weight. The pedagogical model “the plate model” to guide the proportion of various foods on the plates.
3.	The carbon footprint from food production, transportation, and consumption. Be aware of food waste.	Being a conscious consumer. How to read a table of contents on food products. Different types of carbohydrates. The sugar and fiber content in different food products.
4.	The trade-off between healthy foods on one hand and their climate and environmental impact on the other hand. How to use the food guide on climate and environmental impact from WWF^a^. Protein from plant and animal-based foods. Recommended intake of vitamins and minerals.	Be aware of food waste. How products are labeled, including fair trade, sustainable stocks, key hold (healthy foods) on a national and European level.
5.	Being a conscious consumer. How to read a table of contents on food products. Different types of carbohydrates. The sugar and fiber content in different food products. Introduction to citizen science and the use of the Discovery Tool to document facilitators and barriers for a sustainable lifestyle at work.	Recommended intake of vitamins and minerals. Food intake versus supplements. Introduction to citizen science and the use of the Discovery Tool to document facilitators and barriers for a healthy lifestyle at work.
6.	Being a citizen scientist and analyzing photos of facilitators and barriers for a sustainable lifestyle at work. List 3‐4 prioritized barriers at the workplace and advocate for change on an organizational level.	Being a citizen scientist and analyzing photos of facilitators and barriers for a healthy lifestyle at work. List 3‐4 prioritized barriers at the workplace and advocate for change on an organizational level.

a WWF: World Wide Fund for Nature.

### Sustainable Lifestyle Intervention

The participants in the SL arm were instructed to follow the dietary and physical activity recommendations of the World Health Organization (WHO), the Food and Agriculture Organization of the United Nations (FAO), and NNR [39-41]. Additionally, participants were instructed to adopt proenvironmental behaviors in both their work and private lives, defined as actions that minimize environmental harm and combat climate change [42]. For example, increase active transportation to and from work; use active transportation in and around the office workplace; break up prolonged sitting through walk-and-talk meetings; select food items with a low carbon footprint; and purchase organic food instead of conventionally produced food, as seen in Table 1.

### Healthy Lifestyle Intervention

The participants in the HL arm were instructed to follow the dietary recommendations by WHO and NNR 2014, as well as to follow recommendations about the “plate model” (ie, the combination of food on the plate intended to adhere to the recommended intake of nutrients) and to engage in physical activity corresponding to at least 150-300 minutes per week of moderate-vigorous intensity [39,40]; see Table 1.

### Outcome Measures

#### Overview

The primary objective was to assess changes in diet-related CO_2_e as an indicator of food habits with low greenhouse gas emissions, consumption of macro- and micronutrients, fiber, added sugars, physical activity and inactivity levels, and the use of active transportation. Second, the aim was also to evaluate the feasibility of intervention functions within the pilot study by assessing workshop attendance and participants’ self-reported dietary goals, as well as perceived social support, facilitators, and barriers to behavior change.

#### Assessment of Diet, Diet-Related CO_2_e, and Physical Activity

Diet was assessed by the validated and interactive web-based food frequency questionnaire Meal-Q, described in detail elsewhere [43,44], which covers 5 domains of food consumption: (1) habitual intake of food items, dishes, and beverages, including alcoholic beverages; (2) portion sizes; (3) eating behaviors; (4) meal patterns; and (5) food supplements. Data from Meal-Q were linked to the National Food Composition Table from the Swedish National Food Agency to generate the average intake of 50 nutrients per day and per person [45]. Meal-Q was also used to assess the carbon footprint of food consumption, covering the entire “cradle-to-grave” impact of products, and expressed as kilograms of CO_2_e per kilogram of food products (kg CO_2_e per kg) using published Life Cycle Assessment (LCA) data representing the average consumption pattern in Sweden. Each Meal-Q item, including mixed dishes, was linked to an emission factor derived from these LCA data. Composite meals were broken down into their standard recipe components as defined in the Meal-Q nutrient database, and each component was assigned a corresponding CO_2_e value. Participants’ reported intakes were converted into grams per day and multiplied by the relevant emission factors to obtain an average daily CO_2_e estimate per person [46,47].

Physical activity was assessed using the validated and interactive web-based questionnaire Active-Q, described elsewhere [48,49], that covers five domains of activity: (1) level of occupational physical activity; (2) means of transportation to and from daily work or studies; (3) leisure-time activities; (4) exercise; and (5) sleep. Participants reported type, duration, and frequency of various activities in each domain, and data from Active-Q were used to calculate time spent at different activity levels, total metabolic equivalent task (MET), and energy expenditure over a 24-hour period [49,50].

#### Assessment of Attendance Rate, Goal Setting, and Supporting Factors

Participants’ workshop attendance rates were monitored by the research team using attendance lists. During workshop 2, participants filled out printed questionnaires where they formulated individual diet-related goals. These goals were part of the intervention itself and reflected the COM-B–based intervention functions of incentivization, restriction, and self-monitoring. Diet-related goals were selected over physical activity goals because a single focus area is easier for participants to manage than 2; we had a validated assessment method for diet-related carbon footprint, and the impact of greenhouse gas emissions from diet is greater than the impact from physical activity. These goals were self-evaluated along with perceived support from colleagues, managers, family, and the research team, on scales from 0 to 10, where 10 indicated complete fulfillment or good support, at workshops 4 and 6. In addition, participants provided open-ended responses about facilitators and barriers to reaching their goals during workshop 4. These ratings and responses were collected to assess the feasibility of the intervention in terms of supportive factors and to evaluate the intervention functions and educational components.

### Statistical Analysis

Descriptive statistics were used to assess baseline characteristics, workshop attendance rate, goal fulfillment, and social support. Differences in baseline characteristics, goal fulfillment, and social support between the 2 arms were tested using the Student *t* test for numerical variables and a chi-square test for categorical variables. Linear mixed effects models (LMMs) were used to generate mean values and between-arm differences with 95% CIs, and the interaction effect was tested based on the II Wald chi-square test between the time points and arms, with degrees of freedom set to 1 (Multimedia Appendix 1). Finally, the Student *t* test was used to analyze the within-arm mean values and 95% CI between baseline and week 8 separately for each arm, respectively. All participants who were randomized and had answered baseline questionnaires were included in the analysis and analyzed according to intent-to-treat principles, regardless of drop-out, loss to follow-up, or adherence rate. All analyses assumed that numerical variables were normally distributed at the population level [51]. However, clustering was not considered in this study. All quantitative data were analyzed using R statistics version 4.3.2 (October 31, 2023).

### Qualitative Analysis

Printed questionnaires with dietary goals, self-rated goal fulfillment, barriers, and facilitators were transferred to electronic format and analyzed deductively using framework analysis [52]. By reading the goals and narratives independently several times, an initial coding framework was developed, informed by the COM-B model. Thereafter, Excel (Microsoft Corp) spreadsheets were developed, incorporating codes, goals, barriers, and facilitators, resulting in thematic framework matrices. The matrices were filled with goals and participant-specific data by the first author (OHU). As data were compared, the framework was refined and reorganized to accommodate emerging codes and remove those that were no longer relevant. Each goal was coded into categories and analyzed separately for each arm. Moreover, if a goal included 2 dietary aspects, for example, “Eat more fruits and vegetables and increase the proportion of organically produced fruits and vegetables,” it was categorized into 2 different categories. Barriers to and facilitators of behavior change were initially analyzed separately but later combined due to overlap between the SL and HL arms. Thematic codes were then developed as analytic memos to support the creation of themes. Within these broad categories, COM-B mechanisms were identified inductively by interpreting the texts. The organization of the data into COM-B mechanisms and the identification of categories were discussed, refined, and agreed upon by OHU and KB. Final categories were discussed and agreed upon by all researchers, and tables were created to illustrate the categories, codes, COM-B mechanisms, and descriptive quotations.

### Ethical Considerations

The study was approved by the Swedish Ethical Review Authority located in Uppsala (#2021-02309) and conducted in accordance with the guidelines of the Declaration of Helsinki. All research activities strictly followed the regulations set by the Swedish Research Council concerning informed consent and confidentiality. Written informed consent was obtained from all participants prior to any research activities, and trained personnel from the research team were responsible for data management to ensure confidentiality. Moreover, participants were informed of their right to withdraw from the study at any time and to request removal of their data. Participation occurred during working hours and participants were covered by insurance through their respective employers. No financial compensation was provided.

## Results

### Overview

**Figure 2.**
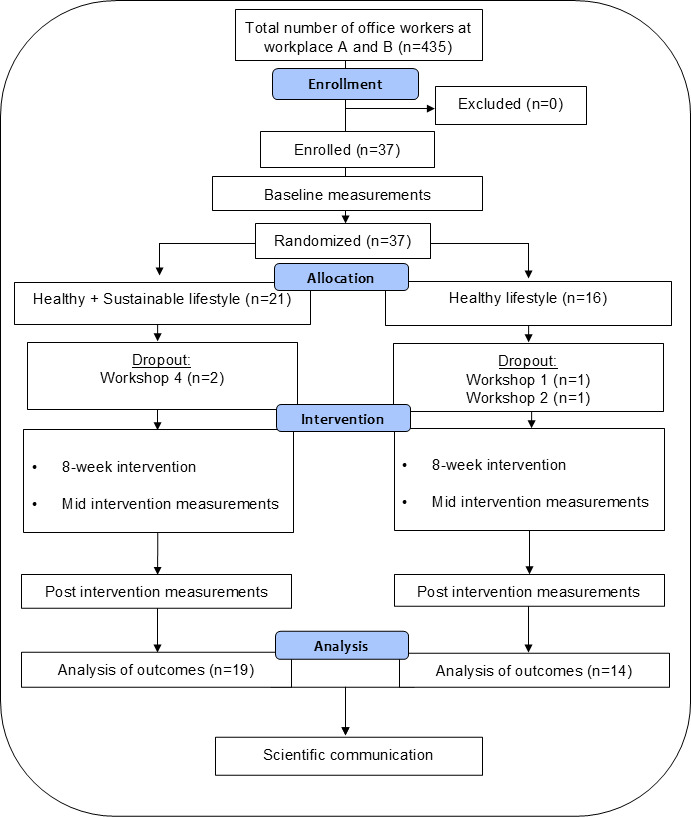
CONSORT (Consolidated Standards of Reporting Trials) flow diagram of participant recruitment and dropout for the Sustainable Office Intervention (SOFIA)–pilot intervention study.

Baseline characteristics of the study participants are shown in Table 2, and there were no statistically significant differences between the 2 arms. The majority of participants were women (28/37, 76%) and had a university degree (33/37, 89%), and all participants were nonsmokers (37/37, 100%). The mean age was 43 years, and the mean BMI was 24. Dropout after randomization was 2 participants in the SL arm and 2 participants in the HL arm; see Figure 2, and the main reason for dropping out was lack of time to participate in the workshops. The overall participant attendance rate at the educational workshops was high, 80% for the SL arm and 78% for the HL arm (Multimedia Appendix 2), and the general reasons for nonattendance were sickness or high workload.

**Table 2.** Baseline demographics of the study population.

Variable	Sustainable lifestyle arm n=21	Healthy lifestyle arm n=16	*P* value^a^
Gender, n (%)			.55
Women	15 (71)	13 (81)	
Men	6 (29)	3 (19)	
Smokers at the time of this writing, n (%)	0 (0)	0 (0)	.55
Highest level of education, n (%)			.55
High school degree	3 (14)	1 (6)	
University degree	18 (86)	15 (94)	
Age (years), mean (SD)	44 (11.4)	43 (8.6)	.83
Weight (kg), mean (SD)	71 (9.2)	69 (9.2)	.62^a^
BMI, mean (SD)	24.5 (2.0)	23.4 (2.1)^b^	.29

a *P* values obtained by Student *t* test for numerical variables and by a chi-square test for categorical variables.

b missing height and weight of one participant who was enrolled but missed baseline anthropometric assessment and later dropped out prior to workshop 1.

**Table 3.** Between-arm differences for daily intake of macro- and micronutrients and carbon footprint per 1000 kcal per day at baseline, 4 weeks, and 8 weeks and within-arm change between baseline assessment and week 8.

Variable	Sustainable lifestyle arm				Healthy lifestyle arm				Between-arm difference^a^ (95% CI)
	n (%)	Mean (SD)	Within-arm change^b^ (95% CI)	*P* value	n (%)	Mean (SD)	Within-arm change^b^ (95% CI)	*P* value	
Total energy intake (kcal)									
Baseline	21 (100)	1772 (669)	—		16 (100)	2004 (605)	—		–232 (–659 to 196)
4 weeks	20 (95)	1712 (770)	—		13 (81)	1721 (617)	—		–112 (–508 to 484)
8 weeks	19 (90)	1581 (683)	—		14 (88)	1617 (441)	—		–36 (–436 to 363)
Baseline versus 8 weeks	18 (86)		–223 (–625 to 179)	.25	13 (81)		–346 (–603 to –90)	.01	
Carbohydrates, E% kcal									
Baseline	21 (100)	41.8 (5.3)	—		16 (100)	41.0 (3.5)	—		0.05 (–2.9 to 3.0)
4 weeks	20 (95)	43.5 (6.5)	—		13 (81)	40.0 (5.3)	—		3.5 (–0.7 to 7.7)
8 weeks	19 (90)	42.9 (6.4)	—		14 (88)	40.9 (4.3)	—		2.0 (–1.7 to 5.8)
Baseline versus 8 weeks	18 (86)		0.7 (–1.4 to 0.02)	.48	13 (81)	–0.8	–0.8 (–2.9 to 1.4)	.45	
Added sugar g per 1000 kcal									
Baseline	21 (100)	16.5 (8.4)	—		16 (100)	19.9 (9.8)	—		–3.5 (–19.7 to 2.7)
4 weeks	20 (95)	18.1 (11.4)	—		13 (81)	20.7 (12.8)	—		–2.5 (–11.6 to 6.4)
8 weeks	19 (90)	18.9 (12.5)	—		14 (88)	21.7 (11.9)	—		–2.8 (–11.5 to 6.0)
Baseline versus 8 weeks	18 (86)		2.2 (–8.6 to 5.2)	.15	13 (81)		0.8 (–1.25 to 2.9)	.40	
Fat, E%									
Baseline	21 (100)	35.8 (4.9)	—		16 (100)	35.7 (3.2)	—		0.1 (2.6 to 2.8)
4 weeks	20 (95)	34.9 (6.0)	—		13 (81)	35.5 (3.3)	—		–0.6 (–3.9 to 2.7)
8 weeks	19 (90)	36.1 (5.9)	—		14 (88)	36.2 (3.0)	—		0.1 (–3.3 to 3.1)
Baseline versus 8 weeks	18 (86)		0.5 (–1.4 to 2.4)	.60	13 (81)		0.24 (–1.7 to 2.1)	.79	
Saturated fat, E%									
Baseline	21 (100)	13.8 (2.0)	—		16 (100)	13.3 (1.5)	—		0.5 (–0.7 to 1.6)
4 weeks	20 (95)	12.6 (3.2)	—		13 (81)	13.7 (1.5)	—		–1.2 (–2.9 to 0.5)
8 weeks	19 (90)	13.4 (4.2)	—		14 (88)	13.4 (2.3)	—		–0.0 (–2.4 to 2.3)
Baseline versus 8 weeks	18 (86)		–0.6 (–2.5 to 1.6)	.63	13 (81)		0.14 (–1.2 to 1.5)	.83	
Protein, E%									
Baseline	21 (100)	17.3 (2.5)	—		16 (100)	16.2 (2.1)	—		1.1 (–0.4 to 2.6)
4 weeks	20 (95)	16.7 (3.4)	—		13 (81)	17.5 (3.2)	—		–0.8 (–3.2 to 1.5)
8 weeks	19 (90)	15.9 (3.5)	—		14 (88)	15.9 (2.0)	—		–0.2 (–2.0 to 2.0)
Baseline versus 8 weeks	18 (86)		–1.3 (–2.7 to 0.1)	.06	13 (81)		0.14 (–7.6 to 1.0)	.75	
Fiber, g per 1000 kcal									
Baseline	21 (100)	2.6 (1.0)	—		16 (100)	2.4 (0.8)	—		0.2 (–0.3 to 0.8)
4 weeks	20 (95)	2.6 (0.8)	—		13 (81)	2.3 (0.6)	—		0.3 (–0.1 to 0.8)
8 weeks	19 (90)	2.6 (1.0)	—		14 (88)	2.4 (0.8)	—		0.2 (0.4 to 0.9)
Baseline versus 8 weeks	18 (86)		–0.25 (–1.8 to 1.3)	.73	13 (81)		−0.08 (–1.2 to 1.0)	.87	
Vitamin C, mg per 1000 kcal									
Baseline	21 (100)	53.5 (15.3)	—		16 (100)	49.1 (21.2)	—		4.4 (–8.4 to 17.3)
4 weeks	20 (95)	68.4 (36.4)	—		13 (81)	64.3 (24.8)	—		4.1 (–17.6 to 25.9)
8 weeks	19 (90)	61.8 (31.9)	—		14 (88)	63.4 (27.8)	—		–1.6 (–22.9 to 19.7)
Baseline versus 8 weeks	18 (86)		8.0 (–2.5 to 20.1)	.12	13 (81)		12.5 (4.8 to 2.0)	.003	
Iron, mg/1000 kcal									
Baseline	21 (100)	5.6 (1.5)	—		16 (100)	4.9 (1.2)	—		0.6 (–0.3 to 1.5)
4 weeks	20 (95)	5.4 (1.2)	—		13 (81)	5.0 (1.2)	—		0.4 (–0.5 to 1.2)
8 weeks	19 (90)	5.6 (1.5)	—		14 (88)	5.0 (1.2)	—		0.6 (–0.4 to 1.5)
Baseline versus 8 weeks	18 (86)		–0.08 (–0.6 to 0.5)	.79	13 (81)		0.2 (9.8 to 5.2)	.16	
Vitamin D, microgram per 1000 kcal									
Baseline	21 (100)	4.3 (1.6)	—		16 (100)	4.7 (1.0)	—		–0.4 (–1.3 to 0.5)
4 weeks	20 (95)	4.8 (1.9)	—		13 (81)	4.6 (1.9)	—		0.2 (–1.2 to 1.6)
8 weeks	19 (90)	4.5 (1.9)	—		14 (88)	4.6 (1.7)	—		–0.1 (–1.4 to 1.2)
Baseline versus 8 weeks	18 (86)		0.2 (–4.5 to 0.9)	.48	13 (81)		0.07 (–7.7 to 9.2)	.85	
Kg CO_2_e per 1000 kcal									
Baseline	21 (100)	2.3 (0.8)	—		16 (100)	2.2 (0.6)	—		0.1 (–0.3 to 0.6)
4 weeks	20 (95)	2.1 (0.7)	—		13 (81)	2.4 (0.5)	—		–0.3 (–0.8 to 0.1]
8 weeks	19 (90)	2.1 (0.8)	—		14 (88)	2.3 (0.5)	—		–0.2 (–0.6 to 0.3)
Baseline versus 8 weeks	18 (86)		–0.16 (–4.3 to 0.11)	.23	13 (81)		0.15 (–6.8 to 3.7)	.15	

a Mean value for between-arm difference at baseline, week 4, and week 8.

b Mean value for within-arm change between baseline and week 8.

### Changes in Dietary Intake Over Time

**Figure 3.**
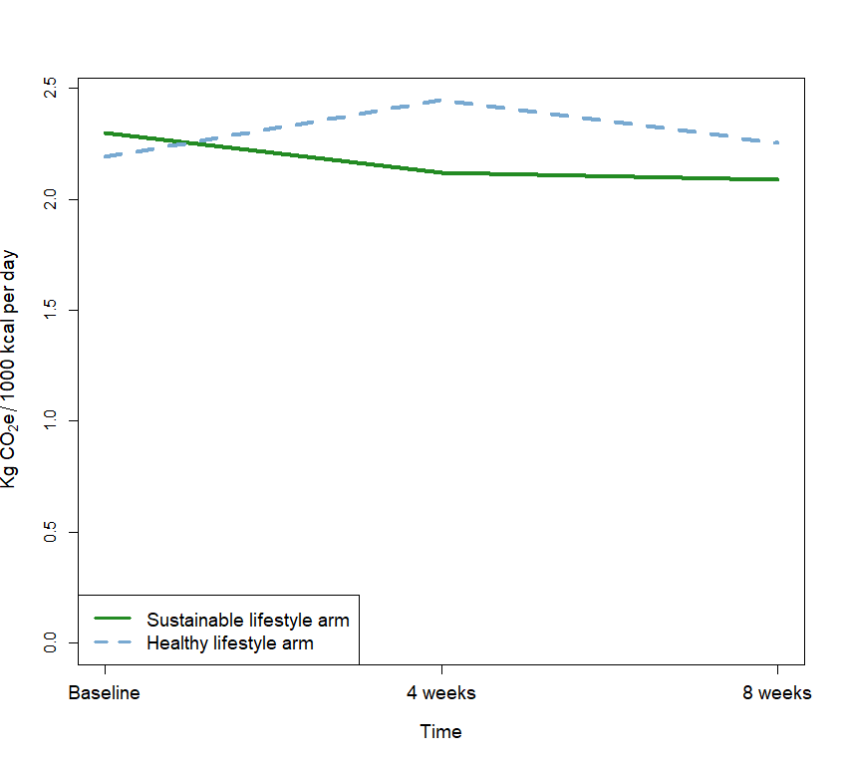
The interaction effect for diet-related kg CO_2_e per 1000 kcal per day between the 2 lifestyle arms over time.

In order to estimate changes in the quality of food intake, nutrients and diet-related carbon footprint (ie, kg CO_2_e) were expressed per 1000 kcal per day in Table 3, and there were no statistically significant differences between the SL arm and the HL at baseline. When testing the change of daily carbon footprint between arms, a significant interaction effect was found between time and intervention arm (*P*=.05), suggesting that the various educational workshops in the SL and HL arms, respectively, had different impacts on carbon footprint over the 8 weeks of intervention, as illustrated in Figure 3. No other between-arm interactions were found for total energy or macro- and micronutrients in the energy-adjusted model (see Multimedia Appendix 1).

However, when analyzing within-arm changes over 8 weeks, the daily intake of vitamin C increased statistically significantly over time in the HL arm, with 12.5 mg per 1000 kcal (*P*=.003). Moreover, total energy decreased statistically significantly over time with 346 kcal per day for the HL arm (*P*=.01), but no other within-arm changes were found for macro- and micronutrients in the energy-adjusted model in Table 3.

In order to estimate changes for the absolute food intake, nutrients, and carbon footprint were expressed per gram or kilogram in MultimediaAppendices 1  3, but no between-arm interactions were found (Multimedia Appendix 1). Analyses of within-arm absolute changes show a statistically significant decrease in the HL arm for total carbohydrate intake by 40.5 grams per day (*P*=.03), total fat by 13 grams per day (*P*=.002), saturated fat by 5 grams (*P*=.03), and iron by 1.7 milligrams (*P*=.04).

Whereas total protein intake was decreased by 17 grams for the SL arm (*P*=.05) and 13.7 grams per day for the HL arm (*P*=.02), and total fiber intake decreased by 5 grams for the SL arm (*P*=.05) and 6 grams per day for the HL arm (*P*=.04) (Multimedia Appendix 3). Moreover, the same nonenergy adjusted model in Multimedia Appendix 3 also showed that total carbon footprint was statistically significantly decreased over time in the SL arm by 0.8 kilograms of CO_2_e per day (*P*=.001). Overall, the SL arm shows more favorable outcomes related to ecological sustainability than the HL arm with respect to total diet-related carbon footprint and a reduction in protein intake over the 8-week intervention period.

### Changes in Physical Activity Outcomes Over Time

There were no between-arm differences at baseline for the SL versus HL arm in MET hours (41.5 vs 41.4) and total sedentary time (h/day; 9.1 vs 8.9). However, the arms differed in baseline active transportation, with the SL arm reporting more minutes per week (43.9 vs 16.8) and higher frequency of active commuting (1.2 vs 0.2 times per week). Additionally, no between-arm interactions were found for the mentioned variables, indicating that educational workshops in the SL and HL arms, respectively, had no impact on physical activity and active transportation (Multimedia Appendix 1). However, when analyzing within-arm changes over 8 weeks, sedentary time decreased statistically significantly in the SL arm (*P*=.03), where total sedentary time decreased by 0.4 hours per day between baseline and 8 weeks (Table 4).

**Table 4.** Differences in daily physical activity and active transportation between arms at baseline, 4 weeks, and 8 weeks, and within-arm change between baseline assessment and Week 8.

Variable	Sustainable lifestyle arm				Healthy lifestyle arm				Between-arm difference^a^ (95% CI)
	n (%)	Mean (SD)	Within-arm change^b^ (95% CI)	*P* value	n (%)	Mean (SD)	Within-arm change^b^ (95% CI)	*P* value	
Total MET^c^ hours (24 h)									
Baseline	21 (100)	41.5 (3.7)	—	—	16 (100)	41.4 (4.6)	—	—	0.1 (–2.9 to 3.9)
4 weeks	20 (95)	42.8 (4.5)	—	—	13 (81)	41.8 (4.0)	—	—	0.9 (–2.1 to 3.9)
8 weeks	19 (90)	42.8 (5.2)	—	—	14 (88)	40.3 (3.5)	—	—	2.5 (–0.6 to 5.6)
Baseline versus 8 weeks	18 (86)	—	1.1 (–0.5 to 2.6)	.17	13 (81)		–0.4 (–2.1 to 1.3)	.64	—
Total sedentary time, h/d									
Baseline	21 (100)	9.1 (1.2)	—	—	16 (100)	8.9 (1.8)	—	—	0.1 (–0.9 to 1.2)
4 weeks	20 (95)	9.9 (1.2)	—	—	13 (81)	9.1 (1.5)	—	—	–0.2 (–1.2 to 0.8)
8 weeks	19 (90)	8.5 (1.0)	—	—	14 (88)	8.9 (1.6)	—	—	–0.5 (–1.4 to 0.5)
Baseline versus 8 weeks	18 (86)	—	–0.4 (–0.85 to –0.04)	.03	13 (81)	—	0.006 (–0.56 to 0.57)	.97	—
Active transportation to and from work, times per week									
Baseline	21 (100)	1.2 (1.8)	—	—	16 (100)	0.2 (0.8)	—	—	1.0 (0.1 to 1.9)
4 weeks	20 (95)	1.5 (2.0)	—	—	13 (81)	0.1 (0.3)	—	—	1.4 (0.5 to 2.4)
8 weeks	19 (90)	1.5 (2.1)	—	—	14 (88)	0.2 (0.8)	—	—	1.3 (0.2 to 2.5)
Baseline versus 8 weeks	18 (86)	—	0.1 (–0.22 to 0.44)	.49	13 (81)		–0.1 (–0.2 to 0.08)	.33	—
Active transportation to and from work, min per week									
Baseline	21 (100)	43.9 (85.8)	—	—	16 (100)	16.8 (57.0)	—	—	27.1 (–21.3 to 75.5)
4 weeks	20 (95)	47.3 (94.3)	—	—	13 (81)	1.1 (4.0)	—	—	46.2 (0.74 to 91.7)
8 weeks	19 (90)	52.7 (107.3)	—	—	14 (88)	14.8 (57.3)	—	—	37.9 (–22.2 to 98.0)
Baseline versus 8 weeks	18 (86)	—	1.5 (–18.9 to 21.9)	.87	13 (81)		–2.1 (–5.3 to 1.0)	.16	—

a Mean value for between-arm difference.

b Mean values for between-arm change at baseline and week 8.

c MET: metabolic equivalent task.

### Diet-Related Goals Set by the Participants

#### Overview

Dietary goals were explored to evaluate potential differences introduced by the different educational approaches in the sustainable arm and the healthy lifestyle arm, and participants’ goals showed a clear alignment with their assigned intervention arm. Some goals overlapped across groups, including goals to consume 500 grams of fruits and vegetables per day and to limit red-meat intake to 500 grams per week. However, participants in the SL arm more often identified goals related to choosing organic foods and increasing the number of vegetarian meals. The following section describes differences and similarities in the individual diet-related goals formulated during workshop 2.

#### Sustainable Diet Goals

Participants in the SL arm formulated 22 different goals, and these were later categorized by the research team into 5 main categories (Multimedia Appendix 4). The first category, increasing intake of nutritionally dense foods, is described by the following quote, “Prioritize nutritionally dense foods and avoid foods with low nutritional value, for example, candy” (SL09), and includes reducing sugar consumption and opting for healthier alternatives, particularly during evenings and weekends. The second category, increasing vegetarian meals, focused on increasing vegetarian meals in the participants’ weekly diet, as one participant wrote, “Eat vegetarian meals at least three times per week” (SL14)*,* where the target number of vegetarian meals ranged from 2 to 3 per week for other participants. Goals in the third category, increasing fruits and vegetables, ranged from specific targets like, “Eat 500 grams of fruits and vegetables per day” (SL17), to more general aims of incorporating fruits and vegetables into every meal and snacks between meals. The fourth category was to increase intake of organically produced foods and revolved around KRAV, and focusing on locally produced items, as described by one participant, “buy[ing] organic foods and products marked KRAV, specifically fruits and vegetables” (SL02). The fifth category, to decrease the intake of red meat, was simply described as “Reduce my intake of red meat to the recommended 500 grams per week, and only eat processed meat once per week” (SL10) as well as incorporating more fish and poultry.

#### Healthy Diet Goals

In total, participants in the HL arm formulated 18 different diet-related goals, and these were later categorized by the research team into 4 main categories (Multimedia Appendix 4). The first category, according to the plate model**,** was described by one participant as “Eat[ing] according to the plate model at lunch and dinner” (HL10) and referred to the model by the Swedish Food Agency to ensure a balanced meal of vegetables, protein, and carbohydrates. The second category, to decrease the intake of red meat to 500 grams per week, involved different goal strategies to lower the intake of red meat, opting for fish and vegetarian options and limiting red meat to 2-3 meals weekly, as stated by one participant.

*Eat less than 500 grams of red meat per week, through removing meat during the lunch and reduce the intake of red meat during other meals, such as dinner.*

[HL12]

The third category by the HL arm was increasing the intake of nutritionally dense foods and describing goals to increase the intake of nutritionally dense foods, including limiting soda intake and prioritizing fruits and vegetables, described as

*eat more nutritionally dense fruits and vegetables, moreover, to avoid carbs such as pasta and rice and eat more whole-grain products*.

[HL22]

The fourth and final category was 500 grams of fruits and vegetables per day and often involved specific targets with a focus on increasing vegetable and fruit intake between main meals, such as the quote

*500 grams of fruits and vegetables per day, two fruits per day, and always salad and vegetables on the plate and more vegetables at dinners.*

[HL23]

### Goal Fulfillment and Perceived Social Support

The individual diet-related goals described above were followed up at workshops 4 and 6, and in general, the self-rated goal fulfillment was high, that is, close to 7, on a scale from 1 to 10; see Table 5. Moreover, the participants perceived support for behavioral change toward a more sustainable or healthy diet, respectively, at workshops 4 and 6, which was rated high, that is, 6.7 or above on a scale from 1 to 10, for the facilitating researcher team, colleagues, family members, and managers, with no differences in ratings between the SL and HL arms.

**Table 5.** Self-rated goal fulfillment and perceived social support for behavior change from family, colleagues, manager, and facilitating researcher team members rated on a 0‐10 scale at workshops 4 and 6.

Variable	Sustainable lifestyle arm, mean (SD)	Healthy lifestyle arm, mean (SD)	*P* value^a^
Workshop 4			
Goal fulfillment	7.0 (1.8)	7.2 (1.2)	.81
Family support	8.3 (1.8)	7.4 (2.7)	.26
Collegial support	7.0 (3.3)	8.0 (3.0)	.29
Manager support	6.7 (3.6)	7.2 (3.0)	.68
Research team support	8.2 (2.8)	8.4 (1.6)	.77
Workshop 6			
Goal fulfillment	7.1 (2.0)	6.4 (1.6)	.32
Family support	7.9 (2.0)	7.4 (2.0)	.56
Collegial support	7.7 (2.4)	8.4 (2.5)	.47
Manager support	7.0 (2.9)	7.5 (2.8)	.67
Research team support	9.3 (1.4)	8.5 (1.6)	.24

a *P *values obtained by Student *t* test.

### Barriers and Facilitators for Behavior Change

Participants in the SL and HL arms identified and formulated perceived barriers and facilitators for behavior change during workshop 4, and these were analyzed by the research team. Despite differences in goal settings (sustainable vs healthy lifestyle), there was considerable overlap, and therefore, all barriers and facilitators merged. In total, 2 themes, 13 categories, and 29 codes were identified, as shown in Table 6. In addition, each category was linked to a specific COM-B mechanism and illustrated by a quote from one of the participants. The first theme, “Barriers to adopt a sustainable or healthy lifestyle” included 8 categories. In brief, high prices of fruits, vegetables, and organic products, worsened by inflation, were a common barrier. Restaurant visits and takeaway food presented challenges due to the lack of transparency in ingredient sourcing and the presence of unhealthy options. Lack of time and planning, influenced by family dynamics and social events, were further complicated by holidays and associated gatherings. Moreover, personal barriers such as difficulty breaking old habits, lack of motivation, and insufficient culinary skills made it challenging for participants to adopt and maintain a sustainable or healthy lifestyle.

**Table 6.** Overview of themes, categories, codes, capability, opportunity, and motivation to support behavior change (COM-B) mechanisms and descriptive quotations for behavior change reported by the participants.

Categories	Codes	COM-B^a^ mechanisms	Descriptive quotation
Theme 1: barriers to adopt a sustainable or healthy lifestyle			
The price on fruits and vegetables and organic products	Price	Physical opportunities	“The price of organically produced products” (SL01). “The price of fruit and vegetables” (SL17).
Restaurant visits and takeaway food	Takeaway Restaurant Eating out	Physical opportunities Reflective motivation Automatic motivation	“When you order takeaway food or eating out you do not always know if the ingredients are organically produced” (SL09).
Lack of time and planning	Lack of time Planning Time pressure	Physical opportunities Psychological capability Reflective motivation	“To some degree, the planning of meals, because of toddlers who don’t eat vegetables, it becomes time-consuming and cumbersome to prepare two dishes” (HL21)
Family support and influence	Children Family	Social opportunities Reflective motivation	“I take care of the grocery shopping, but I need to consider family members who do not eat the same” (SL09) “I have the responsibility for cooking for the whole family and working long days” (HL23)
Holidays such as Christmas and Easter times	Holidays	Social opportunities Reflective motivation Physical opportunities Automatic motivation	“Holiday times and the Easter weekend bring more food and beverages” (SL04).
It is hard to break habits	Old habits	Reflective motivation Automatic motivation	“It is hard to break old habits, and I lack innovative thinking regarding meals” (SL05)
Lack of motivation and discipline	Motivation Laziness Forgetfulness Discipline	Psychological capability Reflective motivation	“My own will, motivation and discipline are what prevent me..” (HL21)
Lack of culinary skills	Not skilled	Physical capability Psychological capability	“My biggest obstacle is that I’m not skilled enough at cooking vegetarian food” (HL15)
Theme 2: facilitators to adopt a sustainable or healthy lifestyle			
Access to lunch restaurants and fruit at work	Restaurant at work Fruit baskets	Physical opportunities	“The lunch restaurant at work facilitates healthy diet through the salad bar; moreover, access to fruit at work” (HL23)
Planning and preparations	Prepare Planning	Reflective motivation Psychological capability Physical opportunities	“Preparing salad for the lunch box for work makes it easier” (SL18)
Access to fruit and vegetables at home	At home Frozen vegetables Fruits I like	Physical opportunities Psychological capability Reflective motivation	“Grocery shopping for the whole week, particularly frozen vegetables” (HL13)
Knowledge of a healthy and sustainable lifestyle	Knowledge	Psychological capability	“Increased knowledge of what is healthy food and how it affects the environment” (SL02)
Healthy lifestyle habits	Exercise Goals Weight	Physical opportunities Reflective motivation	“I have started exercising more, which helps me eat healthier and drink less wine” (HL22)

a COM-B: capability, opportunity, and motivation to support behavior change.

The second theme, “Facilitators to adopt a sustainable or healthy lifestyle” included 5 categories describing facilitators to adopt sustainable or healthy lifestyle habits. In brief, access to healthy options at work, such as salad bars and fruit, made it easier to maintain a healthy diet. Planning and preparation were essential, with participants describing routines like meal planning, grocery shopping for the whole week, and prepping food in advance as perceived facilitators. Moreover, increased knowledge about healthy food and its environmental impact was another important facilitator where the increased understanding of components of a healthy and sustainable diet empowered individuals to make informed food choices. Additionally, creating healthy lifestyle habits, such as exercising more and managing weight and high blood pressure, also supported progress toward personal dietary goals.

## Discussion

### Principal Findings

In this study of 37 generally healthy and normal-weight office workers, cluster-randomized to either a sustainable lifestyle or a healthy lifestyle arm, we found a statistically significant interaction for daily diet-related kg CO_2_e per 1000 kcal from food, indicating that the sustainability education influenced health behaviors differently than the health education over the 8-week period.

Moreover, the total diet-related CO_2_e decreased by 0.8 and 0.4 kg CO_2_e per day in the SL and HL arms, respectively, corresponding to a potential reduction of 292 and 146 kg CO_2_e per person per year. Given that red meat production generates a high carbon footprint whereas plant-based foods generate a low amount, and that high intake of red meat is associated with an increased risk of noncommunicable diseases like obesity, cardiovascular diseases, type 2 diabetes, and certain types of cancer [7-9,53,54], the present approach, which aims to promote both human and planetary health, is worth exploring further. Moreover, considering the office as an arena for promoting a sustainable lifestyle has the potential to reach a large portion of the population.

The reduction of CO_2_e may be the result of the lower intake of energy by 223 and 346 kcal per day for the SL and HL arms, respectively. This is the result of lower intake of total carbohydrate, fat, and, in particular, protein intake between baseline and week 8. However, at the same time, vitamin C increased when expressed as mg per 1000 kcal, indicating that the nutrient density increased, a marker for improved dietary quality and consumption of fruits and vegetables. The energy restriction was not part of the educational workshops and therefore unexpected, but it indicates that the intervention tools and functions were feasible in a society characterized by an unhealthy food environment and overwhelming amounts of foods available consistently. Moreover, a reduction of sedentary time by 0.4 hours per day, corresponding to 2.8 hours per week, was observed in the SL arm. Together, these findings possibly indicate that sustainable and healthy lifestyle practices can both support healthier behaviors in office workers, and be of great relevance, as this population is known to be sedentary at work [55]. This reduction in sedentary time may be partly explained by contextual factors identified in the Our Voice data. Participants in the sustainable lifestyle arm noted barriers to everyday movement, such as limited access to stairwells, and showed stronger motivation to create opportunities for light physical activity during the workday. Between the 2 iterations in data collection, that is, the fall of 2022 and spring of 2023, Office B introduced the option of booking walk-and-talk meetings directly in the digital calendar system. This organizational change likely supported active meetings in line with the message in the sustainable lifestyle to break up prolonged sitting time. Together, these environmental and organizational factors may have contributed to the observed decrease in sedentary time in the sustainable lifestyle arm. However, no changes were found in the time or frequency of active travel, even though the sustainable lifestyle arm took more photos of these elements and initiated a bike-sharing program; see Ullberg et al [36].

This workplace-based educational intervention was grounded in theoretical frameworks targeting dietary and physical activity behaviors [34]. Given that a substantial portion of the adult population spends considerable time at work daily, office-based interventions allow many individuals to participate simultaneously [17,56]. From the employees’ perspective, these interventions are convenient, easily accessible, and foster interaction and mutual support for behavior change efforts [57]. By targeting capabilities, opportunities, and motivation based on the Behavioral Change Wheel and COM-B [58], we enhance the intervention’s effectiveness in the office context. Moreover, implementing the COM-B mechanisms in combination with the socioecological model increases the understanding of behaviors in relation to the workplace environment [31,32].

The goals set by participants at the beginning of the intervention aligned well with each intervention arm. Although there was some overlap, such as the goal of consuming 500 grams of fruits and vegetables per day and 500 grams of red meat per week, the SL arm included more goals related to organic foods and increasing the number of vegetarian meals per week, as compared to the HL arm. Despite these differences, the barriers and facilitators described were quite similar, likely due to the inherent complexity of changing one’s diet, whether the change is for health or sustainability reasons [59,60].

Both arms had approximately an 80% attendance rate at the educational workshops, and participants reported overall strong social support from colleagues, researchers, and managers. In addition, the goals formulated within each arm aligned well with the content of the corresponding educational workshops. Taken together, these findings indicate that key components were implemented as intended within this pilot. However, the intervention is time-consuming and places a burden on the company’s already overbooked conference rooms and participants’ schedules, making it hard to be compatible with the existing system and to allocate enough infrastructure support. Therefore, future iterations of the SOFIA study may incorporate digital education sessions, use interactive online course modules, and involve the human resources department to a greater extent. Additionally, the study participants identified potential barriers and facilitators to healthy and sustainable lifestyles outside or beyond the context of the workplace, such as planning, motivation, takeaway food, and the complexities of family dinners. These factors should be highlighted in future interventions in connection to COM-B mechanisms, such as social and physical opportunities, automatic and reflective motivation, and psychological capabilities to further support behavioral change based on the Behavioral Change Wheel [58].

### Limitations

This is a pilot study with a small sample size with limited statistical power to detect the intervention’s effects on nutritional and physical activity variables and may be subjected to random bias. However, we did find an intervention effect for daily diet-related kg CO_2_e per 1000 kcal and a reduction in sedentary time in the SL arm, indicating that a future scaling of the present study is reasonable. However, the small sample size of predominantly women indicates that a more effective recruitment strategy is necessary for a full-scale trial. The use of a validated food and physical activity questionnaire (Meal-Q and Active-Q) to assess dietary intake, carbon footprint, and physical activity was a strength [44,49], but self-reported data may still introduce social desirability and recall bias, resulting in potential over- or underestimation of the true dietary intake and level of physical activity. To overcome these issues, CO_2_e and nutrients were expressed per 1000 kcal and MET-hours adjusted to per 24 hours to highlight the quality and not only the absolute quantity.

The use of cluster randomization offered several strengths and limitations. By targeting geographically separated groups, the intervention minimizes cross-group influence. However, this method has drawbacks, as individuals within a cluster often resemble each other more than those in different clusters, requiring a larger sample size to account for the lack of variation as well as imbalances between groups. Despite these challenges, the SOFIA study design takes advantage of social influence within groups, as colleagues can support each other, potentially enhancing the intervention’s effects. Also, the upscaled version of the SOFIA study should incorporate intracluster correlation coefficients to provide a more accurate assessment by allowing for appropriate adjustments in the statistical models.

This study has been retrospectively registered as a full-scale clinical trial on ClinicalTrials.gov (ID no. NCT06698094) and reports data according to the CONSORT checklist (Checklist 1). By registering and publishing a study protocol and additional findings from the SOFIA pilot phase [36], we have maintained transparency about our hypotheses and planned outcomes, which guides and strengthens the development of the SOFIA study [34]. Moreover, it addresses issues such as p-hacking and selective reporting of variables [61].

### Comparison With Prior Work

A previous Swedish study using Meal-Q, including 5364 well-educated adults aged 18‐45 years, showed that the carbon footprint from their food consumption was, on average, 4.7 kg CO_2_e per person per day, which corresponds to 1.7 tons of CO_2_e per person and per year [62]. At the end of the present intervention, the SL arm had a mean CO_2_e per person and day of 3.0 kg, corresponding to 1 ton per year, while the HL arm had a mean CO_2_e per person and per day of 3.6 kg, corresponding to 1.3 tons of CO_2_e per person per year, in line with lacto-ovo-vegetarian and pescetarian diets in other studies [63]. This suggests that relative minor dietary changes can result in CO_2_e emissions from food close to 1 ton per person per year, a target number set by the Swedish Environmental Protection Agency back in 2015 [64].

However, this study is one of the few that have investigated CO_2_e emissions from food as the primary outcome in an intervention study and the only workplace-based intervention known to the authors. A recent randomized controlled trial in Sweden assessed the effects of an app-based educational intervention on the dietary intake of individuals with type 2 diabetes. The 12-week intervention provided participants with recommendations from the Swedish Food Agency, hypothesizing that healthier food choices would also reduce CO_2_e emissions per person per day. However, the ad hoc analyses showed no difference in CO_2_e emissions between the intervention and control groups [65], and this may be explained by the lack of information about diet-related carbon footprint during the intervention.

A 2-armed multicomponent behavioral randomized controlled trial in the United Kingdom used meat substitutes, information, and social support among adults and resulted in statistically significant reductions in CO_2_e after 8 weeks. The intervention group reported emissions of 31.7 kg per week (4.5 kg/d), compared to 40.3 kg/wk (5.7 kg/d) in the control group at the end of the intervention [66]. Moreover, in a pilot study from the United States, seminars in academic campus courses were used to explore food systems and environmental sustainability. Students in the intervention group lowered their daily dietary carbon footprint by an estimated 0.5 kg CO_2_e per 2000 kcal per day [67]. Another study compared a sustainable education cluster with those in a control cluster, and at the 6-month follow-up, the dietary footprints for students in the sustainable education cluster were 4.15 kg CO_2_e per day for female students and 5.72 kg CO_2_e per day for male students, compared to 4.94 kg CO_2_e per day for females and 6.95 kg CO_2_e per day for males in the control cluster [68].

Comparisons with previous work conclude that interventions should specifically target sustainable lifestyle aspects, such as reducing meat intake through substitutes or increased plant-based foods, compared to general health recommendations when aiming at reducing carbon footprint from food. Interventions should also include social support, goal-setting, self-monitoring, action planning, prompts and cues, recipes, culinary skill tips, and education focused on the environmental impact of food production. Compared to the present study, participants in previous studies have generally higher levels of diet-related CO_2_e per day at the end of each intervention, levels that were closer to those of our participants’ levels at baseline. This could be due to the use of a different LCA calculation and allocation, making it challenging to quantify and compare reduction in CO_2_e, or it may reflect that our study population had different food habits due to high health literacy since they were well educated, had normal BMI, were nonsmokers, and were physically active, potentially resulting in lower meat consumption at baseline. However, protein intake, particularly meat consumption, is high in a typical Western diet [69] and substantially contributes to diet-related CO_2_e. Therefore, our findings are likely generalizable to office workers in countries where a Western diet is common.

### Conclusions

This study suggests that healthy lifestyle recommendations embedded in sustainable development can reduce participants’ food-related CO_2_e in a workplace context. Additionally, the present study indicates that adopting a sustainable lifestyle has a small but significant effect on the intake of key nutrients such as vitamin C, and reduction of total sitting time, indicating that a sustainable lifestyle is a healthy lifestyle. Moreover, the goals set by participants aligned well with the content of the corresponding educational workshops, and the workshop attendance rate was high (80%), indicating good feasibility of study procedures. Given the complex global challenges of climate change, environmental degradation, and the epidemic of metabolic diseases, future intervention studies about healthy behavior should therefore be integrated with sustainable development.

## Supplementary material

10.2196/82061 Multimedia Appendix 1 Table with interaction effects of nutrients, CO_2_e, and physical activity variables.

10.2196/82061 Multimedia Appendix 2 Participant workshop attendance rate.

10.2196/82061 Multimedia Appendix 3 Table with absolute nutrient and CO_2_e variables.

10.2196/82061 Multimedia Appendix 4 Table describing dietary goals, themes, categories, and codes.

10.2196/82061 Checklist 1 CONSORT checklist.

## Abbreviations

CO_2_e: carbon dioxide equivalent
COM-B: capability, opportunity, and motivation to support behavior change
CONSORT: Consolidated Standards of Reporting Trials
FAO: Food and Agriculture Organization of the United Nations
HL: healthy lifestyle
LCA: Life Cycle Assessment
LMM: linear mixed effects model
MET: metabolic equivalent task
NNR: Nordic Nutrition Recommendations
SL: sustainable lifestyle
SOFCO: Concepts for the Sustainable Office of the Future
SOFIA: Sustainable Office Intervention
WHO: World Health Organization

## References

[1] Ding D, Lawson KD, Kolbe-Alexander TL (2016). The economic burden of physical inactivity: a global analysis of major non-communicable diseases. Lancet.

[2] Rachmah Q, Martiana T, Mulyono M (2021). The effectiveness of nutrition and health intervention in workplace setting: a systematic review. J Public Health Res.

[3] Grimani A, Aboagye E, Kwak L (2019). The effectiveness of workplace nutrition and physical activity interventions in improving productivity, work performance and workability: a systematic review. BMC Public Health.

[4] Schultz AB, Chen CY, Edington DW (2009). The cost and impact of health conditions on presenteeism to employers: a review of the literature. Pharmacoeconomics.

[5] Jones CM, Kammen DM (2011). Quantifying carbon footprint reduction opportunities for U.S. households and communities. Environ Sci Technol.

[6] (2022). Climate change 2022: mitigation of climate change. IPCC.

[7] Satija A, Hu FB (2018). Plant-based diets and cardiovascular health. Trends Cardiovasc Med.

[8] Waddell IS, Orfila C (2023). Dietary fiber in the prevention of obesity and obesity-related chronic diseases: from epidemiological evidence to potential molecular mechanisms. Crit Rev Food Sci Nutr.

[9] World Health Organization (2022). Global status report on physical activity 2022. https://www.who.int/publications/i/item/9789240059153.

[10] Gimeno-Ruiz S, Guallar-Castillón P, López-García E (2025). Metabolic and environmental benefits of following the healthy and sustainable dietary recommendations for the Spanish population: the AWHS study. Nutrients.

[11] Thomas MS, Calle M, Fernandez ML (2023). Healthy plant-based diets improve dyslipidemias, insulin resistance, and inflammation in metabolic syndrome. A narrative review. Adv Nutr.

[12] Bourne JE, Sauchelli S, Perry R (2018). Health benefits of electrically-assisted cycling: a systematic review. Int J Behav Nutr Phys Act.

[13] Abu-Omar K, Chevance G, Tcymbal A, Gelius P, Messing S (2023). Physical activity promotion, human and planetary health – a conceptual framework and suggested research priorities. J Clim Change Health.

[14] Bernard P, Chevance G, Kingsbury C (2021). Climate change, physical activity and sport: a systematic review. Sports Med.

[15] Bui LP, Pham TT, Wang F (2024). Planetary Health Diet Index and risk of total and cause-specific mortality in three prospective cohorts. Am J Clin Nutr.

[16] Stubbendorff A, Ericson U, Hallström E, Samuelsson J, Sonestedt E, Ibsen DB (2026). Nutritional adequacy of the EAT-Lancet diet: a Swedish population-based cohort study. Lancet Planet Health.

[17] (2015). The work environment 2015. https://www.av.se/globalassets/filer/statistik/arbetsmiljon-2015/arbetsmiljostatistik-arbetsmiljon-2015-rapport-2016-2.pdf.

[18] (2021). Labour force survevs (LFS). SCB Statistics Sweden.

[19] Glympi A, Chasioti A, Bälter K (2020). Dietary interventions to promote healthy eating among office workers: a literature review. Nutrients.

[20] Hyży A, Jaworski M, Cieślak I, Gotlib-Małkowska J, Panczyk M (2023). Improving eating habits at the office: an umbrella review of nutritional interventions. Nutrients.

[21] Virtanen M, Lallukka T, Elovainio M, Steptoe A, Kivimäki M (2025). Effectiveness of workplace interventions for health promotion. Lancet Public Health.

[22] Lock M, Post D, Dollman J, Parfitt G (2021). Efficacy of theory-informed workplace physical activity interventions: a systematic literature review with meta-analyses. Health Psychol Rev.

[23] Dabkowski E, Porter JE, Barbagallo M, Prokopiv V, Snell C, Missen K (2023). A systematic literature review of workplace physical activity programs: an exploration of barriers and enabling factors. Cogent Psychol.

[24] Marin-Farrona M, Wipfli B, Thosar SS (2023). Effectiveness of worksite wellness programs based on physical activity to improve workers’ health and productivity: a systematic review. Syst Rev.

[25] Peñalvo JL, Sagastume D, Mertens E (2021). Effectiveness of workplace wellness programmes for dietary habits, overweight, and cardiometabolic health: a systematic review and meta-analysis. Lancet Public Health.

[26] Gea Cabrera A, Caballero P, Wanden-Berghe C, Sanz-Lorente M, López-Pintor E (2021). Effectiveness of workplace-based diet and lifestyle interventions on risk factors in workers with metabolic syndrome: a systematic review, meta-analysis and meta-regression. Nutrients.

[27] Goetzel RZ, Henke RM, Tabrizi M (2014). Do workplace health promotion (wellness) programs work?. J Occup Environ Med.

[28] Hutchinson AD, Wilson C (2012). Improving nutrition and physical activity in the workplace: a meta-analysis of intervention studies. Health Promot Int.

[29] Wadi NM, Cheikh K, Keung YW, Green R (2024). Investigating intervention components and their effectiveness in promoting environmentally sustainable diets: a systematic review. Lancet Planet Health.

[30] Abdin S, Welch RK, Byron-Daniel J, Meyrick J (2018). The effectiveness of physical activity interventions in improving well-being across office-based workplace settings: a systematic review. Public Health (Fairfax).

[31] van Kasteren YF, Lewis LK, Maeder A (2020). Office-based physical activity: mapping a social ecological model approach against COM-B. BMC Public Health.

[32] Michie S, van Stralen MM, West R (2011). The behaviour change wheel: a new method for characterising and designing behaviour change interventions. Implement Sci.

[33] To QG, Chen TTL, Magnussen CG, To KG (2013). Workplace physical activity interventions: a systematic review. Am J Health Promot.

[34] Bälter K, King AC, Fritz J, Tillander A, Halling Ullberg O (2024). Sustainable lifestyle among office workers (the SOFIA Study): protocol for a cluster randomized controlled trial. JMIR Res Protoc.

[35] Appel-Meulenbroek R (2016). Modern offices and new ways of working studied in more detail. J Corp Real Estate.

[36] Ullberg OH, Toivanen S, King AC, Bälter K (2024). Using citizen science to explore barriers and facilitators for healthy and sustainable lifestyles in office environments. Health Place.

[37] Buman MP, Winter SJ, Sheats JL (2013). The stanford healthy neighborhood discovery tool. Am J Prev Med.

[38] Michie S, Richardson M, Johnston M (2013). The behavior change technique taxonomy (v1) of 93 hierarchically clustered techniques: building an international consensus for the reporting of behavior change interventions. Ann Behav Med.

[39] NNR (2014). Nordic Nutrition Recommendations 2012: Integrating Nutrition and Physical Activity.

[40] Bull FC, Al-Ansari SS, Biddle S (2020). World Health Organization 2020 guidelines on physical activity and sedentary behaviour. Br J Sports Med.

[41] FAO (2019). Sustainable healthy diets: guiding principles. https://www.who.int/publications/i/item/9789241516648.

[42] Steg L, Vlek C (2009). Encouraging pro-environmental behaviour: an integrative review and research agenda. J Environ Psychol.

[43] Christensen SE, Möller E, Bonn SE (2013). Two new meal- and web-based interactive food frequency questionnaires: validation of energy and macronutrient intake. J Med Internet Res.

[44] Christensen SE, Möller E, Bonn SE (2014). Relative validity of micronutrient and fiber intake assessed with two new interactive meal- and web-based food frequency questionnaires. J Med Internet Res.

[45] (2023). Nutrients (the swedish food composition database). Swedish Food Agency.

[46] Baumann H, Tillman AM (2004). The Hitch Hiker’s Guide to LCA: An Orientation in Life Cycle Assessment Methodology and Application.

[47] Sjörs C, Raposo SE, Sjölander A, Bälter O, Hedenus F, Bälter K (2016). Diet-related greenhouse gas emissions assessed by a food frequency questionnaire and validated using 7-day weighed food records. Environ Health.

[48] Bonn SE, Bergman P, Trolle Lagerros Y, Sjölander A, Bälter K (2015). A validation study of the web-based physical activity questionnaire Active-Q against the GENEA accelerometer. JMIR Res Protoc.

[49] Bonn SE, Trolle Lagerros Y, Christensen SE (2012). Active-Q: validation of the web-based physical activity questionnaire using doubly labeled water. J Med Internet Res.

[50] Ainsworth BE, Haskell WL, Herrmann SD (2011). 2011 compendium of physical activities: a second update of codes and MET values. Med Sci Sports Exerc.

[51] Jiang J (2007). Linear and Generalized Linear Mixed Models and Their Applications [Electronic Resource].

[52] Gale NK, Heath G, Cameron E, Rashid S, Redwood S (2013). Using the framework method for the analysis of qualitative data in multi-disciplinary health research. BMC Med Res Methodol.

[53] Wall CR, Stewart AW, Hancox RJ (2018). Association between frequency of consumption of fruit, vegetables, nuts and pulses and BMI: analyses of the International Study of Asthma and Allergies in Childhood (ISAAC). Nutrients.

[54] (2018). About our cancer prevention. World Cancer Research Fund.

[55] Prince SA, Elliott CG, Scott K, Visintini S, Reed JL (2019). Device-measured physical activity, sedentary behaviour and cardiometabolic health and fitness across occupational groups: a systematic review and meta-analysis. Int J Behav Nutr Phys Act.

[56] Hallman DM, Januario LB, Mathiassen SE, Heiden M, Svensson S, Bergström G (2021). Working from home during the COVID-19 outbreak in Sweden: effects on 24-h time-use in office workers. BMC Public Health.

[57] Abood DA, Black DR, Feral D (2003). Nutrition education worksite intervention for university staff: application of the health belief model. J Nutr Educ Behav.

[58] Michie S, Atkins L, West R (2014). The Behaviour Change Wheel A Guide to Designing Interventions.

[59] Timlin D, McCormack JM, Simpson EE (2021). Using the COM-B model to identify barriers and facilitators towards adoption of a diet associated with cognitive function (MIND diet). Public Health Nutr.

[60] Tsofliou F, Vlachos D, Hughes C, Appleton KM (2022). Barriers and facilitators associated with the adoption of and adherence to a Mediterranean style diet in adults: a systematic review of published observational and qualitative studies. Nutrients.

[61] Stefan AM, Schönbrodt FD (2023). Big little lies: a compendium and simulation of *p*-hacking strategies. R Soc Open Sci.

[62] Bälter K, Sjörs C, Sjölander A, Gardner C, Hedenus F, Tillander A (2017). Is a diet low in greenhouse gas emissions a nutritious diet? - Analyses of self-selected diets in the LifeGene study. Arch Public Health.

[63] Scarborough P, Appleby PN, Mizdrak A (2014). Dietary greenhouse gas emissions of meat-eaters, fish-eaters, vegetarians and vegans in the UK. Clim Change.

[64] (2015). Hållbara konsumtionsmönster. https://www.naturvardsverket.se/publikationer/6600/hallbara-konsumtionsmonster/.

[65] Pitt S, Sjöblom L, Bälter K, Trolle Lagerros Y, Bonn SE (2023). The effect of an app-based dietary intervention on diet-related greenhouse gas emissions - results from a randomized controlled trial. Int J Behav Nutr Phys Act.

[66] Bianchi F, Stewart C, Astbury NM, Cook B, Aveyard P, Jebb SA (2022). Replacing meat with alternative plant-based products (RE-MAP): a randomized controlled trial of a multicomponent behavioral intervention to reduce meat consumption. Am J Clin Nutr.

[67] Malan H, Amsler Challamel G, Silverstein D (2020). Impact of a scalable, multi-campus “Foodprint” seminar on college students’ dietary intake and dietary carbon footprint. Nutrients.

[68] Jay JA, D’Auria R, Nordby JC (2019). Reduction of the carbon footprint of college freshman diets after a food-based environmental science course. Clim Change.

[69] Fanzo J, Rudie C, Sigman I (2022). Sustainable food systems and nutrition in the 21st century: a report from the 22nd annual Harvard Nutrition Obesity Symposium. Am J Clin Nutr.

