# Supplementary material for: A Sustainable Lifestyle Intervention Among Office Workers: Cluster Randomized Pilot and Feasibility Study
Source: JMIR Form Res. 2026 May 7;10:e82061. doi: 10.2196/82061 (PMC13152203; doi:10.2196/82061)
Supplement: Multimedia Appendix 1 [file formative-v10-e82061-s001.docx]

**Multimedia Appendix 1**

Test results of interaction effects sustainable lifestyle vs. healthy lifestyle over the 8-weeks using Chi-squared test from the Linear mixed-effects models (LMMs).

| Variable | Chi-squared (χ2) | Degree of freedom | *P*-value |
| --- | --- | --- | --- |
| Total energy intake, Kcal | 0.5 | 1 | 0.5 |
| Carbohydrates, E% | 1.5 | 1 | 0.2 |
| Carbohydrates, g | 1.04 | 1 | 0.3 |
| Added sugar g/1000kcal | 0.2 | 1 | 0.6 |
| Added sugar, g | 1.0 | 1 | 0.3 |
| Fat, E% | 0.001 | 1 | 0.1 |
| Fat, g | 0.4 | 1 | 0.5 |
| Saturated fat, E% | 0.4 | 1 | 0.5 |
| Saturated fat, g | 0.2 | 1 | 0.7 |
| Protein, E% | 2.5 | 1 | 0.1 |
| Protein, g | 0.07 | 1 | 0.8 |
| Fiber, g/1000 kcal | 0.01 | 1 | 0.9 |
| Fiber, g | 0.9 | 1 | 0.8 |
| Vitamin C, mg/1000kcal | 0.2 | 1 | 0.6 |
| Vitamin C, mg | 0.3 | 1 | 0.5 |
| Vitamin D, Microgram/1000kcal | 0.2 | 1 | 0.6 |
| Vitamin D, Microgram | 0.4 | 1 | 0.5 |
| Iron, mg/1000kcal | 0.5 | 1 | 0.4 |
| Iron, mg | 0.2 | 1 | 0.7 |
| CO_2_e/kg/1000kcal/day | 4.0 | 1 | 0.046 |
| CO_2_e/kg/day | 1.1 | 1 | 0.3 |
| Total MET hours, 24h | 2.7 | 1 | 0.1 |
| Total sedentary time, h/day | 2.1 | 1 | 0.1 |
| Active transportation to and from work, times/week | 1.4 | 1 | 0.2 |
| Active transportation to and from work, min/week | 0.4 | 1 | 0.5 |
