# Supplementary material for: A Sustainable Lifestyle Intervention Among Office Workers: Cluster Randomized Pilot and Feasibility Study
Source: JMIR Form Res. 2026 May 7;10:e82061. doi: 10.2196/82061 (PMC13152203; doi:10.2196/82061)

**Multimedia Appendix 2:** Attendance rate at educational workshops 1 to 6, for the sustainable lifestyle arm and respectively.


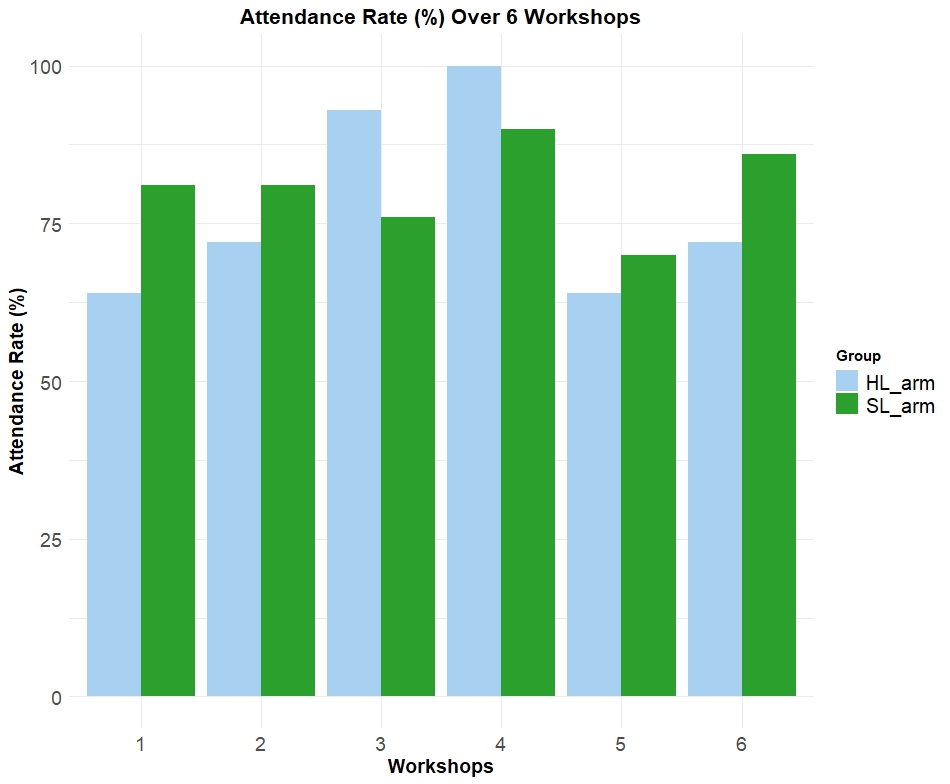

Supplement: Multimedia Appendix 2 [file formative-v10-e82061-s002.docx]
