# Supplementary material for: A Sustainable Lifestyle Intervention Among Office Workers: Cluster Randomized Pilot and Feasibility Study
Source: JMIR Form Res. 2026 May 7;10:e82061. doi: 10.2196/82061 (PMC13152203; doi:10.2196/82061)
Supplement: Multimedia Appendix 3 [file formative-v10-e82061-s003.docx]

**Multimedia Appendix 3:** Between arm differences for daily intake of macro- and micronutrients and carbon footprint per day at baseline, 4 weeks and 8 weeks, and within arm change between baseline assessment and week 8.

|  | Sustainable lifestyle | |  |  | Healthy lifestyle | |  |  |  |
| --- | --- | --- | --- | --- | --- | --- | --- | --- | --- |
|  | N | Mean (SD) | Within-arm change^a^ [95%CI] | *P*-value | N | Mean (SD) | Within-arm change^a^ [95%CI] | *P*-value | Between-arm difference ^b^ [95%CI] |
| Total energy intake, Kcal |  |  |  |  |  |  |  |  |  |
| Baseline | 21 | 1772 (669) |  |  | 16 | 2004 (605) |  |  | -232 [-659, 196) |
| 4 weeks | 20 | 1712 (770) |  |  | 13 | 1721 (617) |  |  | -112 [-508, 484] |
| 8 weeks | 19 | 1581 (682) |  |  | 14 | 1617 (441) |  |  | -36 [-436, 363] |
| Within arm change baseline vs. 8 weeks | 18 |  | -223 [-625, 179] | .25 | 13 |  | -346 [-603, -90] | .01 |  |
| Carbohydrates, g |  |  |  |  |  |  |  |  |  |
| Baseline | 21 | 186.3 (75.0) |  |  | 16 | 211.0 (70.1) |  |  | -24.8 [-73.5, 23.9] |
| 4 weeks | 20 | 186.3 (17.8) |  |  | 13 | 173 (67.9) |  |  | 12.4 [-43.5, 68.4] |
| 8 weeks | 19 | 171.2 (83.7) |  |  | 14 | 165.8 (48.7) |  |  | 5.4 [-41.9, 52.8] |
| Within arm change baseline vs. 8 weeks | 18 |  | -19.6 [-63.5, 24.3] | .36 | 13 |  | -40.5 [-75.8, -5.18] | .03 |  |
| Added sugar g |  |  |  |  |  |  |  |  |  |
| Baseline | 21 | 23.8 (16.2) |  |  | 16 | 34.3 (18.3) |  |  | -10 [-22.3, 1.3] |
| 4 weeks | 20 | 24.4 (17.8) |  |  | 13 | 33.3 (27.1) |  |  | -8 [-26.3, 9.3] |
| 8 weeks | 19 | 29.8 (43.1) |  |  | 14 | 31.0 (20.4) |  |  | -1.1 [-24.3, 22.0] |
| Within arm change baseline vs. 8 weeks | 18 |  | 6.2 [-13.4, 26.0] | .51 | 13 | -3.0 | -3.0 [-6.7, 0.63] | .09 |  |
| Fat, g |  |  |  |  |  |  |  |  |  |
| Baseline | 21 | 70.7 (32.4) |  |  | 16 | 79 (22.9) |  |  | -8.3 [-26.9, 10.1] |
| 4 weeks | 20 | 67.8 (35.7) |  |  | 13 | 68.3 (25) |  |  | -0.4 [-22.0, 21.1] |
| 8 weeks | 19 | 64.7 (35.5) |  |  | 14 | 65.2 (18.3) |  |  | -0.4 [-19.9, 19.0] |
| Within arm change baseline vs. 8 weeks | 18 |  | -7.0 [-28.0, 14.0] | .49 | 13 |  | -13.0 [-20.5, -5.4] | .002 |  |
| Saturated fat, g |  |  |  |  |  |  |  |  |  |
| Baseline | 21 | 27.5 (13.8) |  |  | 16 | 29.7 (9.6) |  |  | -2.1 [-10.0, 5.6] |
| 4 weeks | 20 | 23.7 (10.5) |  |  | 13 | 26.7 (10.8) |  |  | -3.0 [-10.8, 4.8] |
| 8 weeks | 19 | 24.9 (20.9) |  |  | 14 | 24.3 (8.4) |  |  | 0.6 [-10.3, 11.5] |
| Within arm change baseline vs. 8 weeks | 18 |  | -3.2 [-15.6, 9.2] | .59 | 13 |  | -5.0 [-9.0, -0.4] | .03 |  |
| Protein, g |  |  |  |  |  |  |  |  |  |
| Baseline | 21 | 75 (26.1) |  |  | 16 | 82.1 (31.7) |  |  | -6.2 [-26.2, 13.7] |
| 4 weeks | 20 | 68.7 (25.9) |  |  | 13 | 74.3 (25.8) |  |  | -5.6 [-24.5, 13.4] |
| 8 weeks | 19 | 59.8 (18.6) |  |  | 14 | 64.2 (18.3) |  |  | -4.4 [-17.7, 8.8] |
| Within arm change baseline vs 8 weeks | 18 |  | -17.0 [-28.1, -6.0] | .05 | 13 |  | -13.7 [-24.6, 2.7] | .02 |  |
| Fiber, g |  |  |  |  |  |  |  |  |  |
| Baseline | 21 | 24.7 (16.8) |  |  | 16 | 24.8 (13.9) |  |  | -0 [-10.5, 10.1] |
| 4 weeks | 20 | 23.6 (16.1) |  |  | 13 | 19.1 (6.7) |  |  | 4.5 [-3.8, 12.8] |
| 8 weeks | 19 | 20.5 (10.5) |  |  | 14 | 19.2 (7.8) |  |  | 1.3 [-5.2, 7.9] |
| Within arm change baseline vs. 8 weeks | 18 |  | -5.0 [-10.2, -0.2] | .05 | 13 |  | -6.0 [-11.1, -0.4] | .04 |  |
| Vitamin C, mg |  |  |  |  |  |  |  |  |  |
| Baseline | 21 | 96.5 (52.3) |  |  | 16 | 98.3 (57.4) |  |  | -1.7 [-39.1, 35.7] |
| 4 weeks | 20 | 114.7 (98.7) |  |  | 13 | 107.6 (51.3) |  |  | 7.1 [-46.5, 60.7] |
| 8 weeks | 19 | 85.8 (38.2) |  |  | 14 | 101.1 (47.1) |  |  | -15.4 [-47.0, 16.3] |
| Within arm change baseline vs. 8 weeks | 18 |  | -12 [-27.2, 3.7] | .12 | 13 |  | -0.7 [-23.5, 22.1] | .09 |  |
| Iron, mg |  |  |  |  |  |  |  |  |  |
| Baseline | 21 | 10.4 (6.7) |  |  | 16 | 10.3 (4.9) |  |  | 0.04 [-8.5, 3.9] |
| 4 weeks | 20 | 9.4 (4.7) |  |  | 13 | 8.7 (3.6) |  |  | 0.7 [-2.3, 3.6] |
| 8 weeks | 19 | 9.4 (6.4) |  |  | 14 | 8.1 (2.9) |  |  | 1.3 [-2.1, 4.7] |
| Within arm change baseline vs. 8 weeks | 18 |  | -1.3 [-5.1, 2.4] | .47 | 13 |  | -1.7 [-3.4, -0.06] | .04 |  |
| Vitamin D, Microgram |  |  |  |  |  |  |  |  |  |
| Baseline | 21 | 7.6 (3.9) |  |  | 16 | 9.5 (4.1) |  |  | -1.9 [-4.6, 0.8] |
| 4 weeks | 20 | 8.3 (5.0) |  |  | 13 | 7.5 (2.3) |  |  | 0.8 [-1.9, 3.4] |
| 8 weeks | 19 | 6.8 (3.3) |  |  | 14 | 7.5 (4.4) |  |  | -0.8 [-3.6, 2.2] |
| Within arm change baseline vs. 8 weeks | 18 |  | -0.8 [-2.9, 1.3] | .42 | 13 |  | -1.3 [-4.1, 1.5] | .32 |  |
| Kg CO_2_e/day |  |  |  |  |  |  |  |  |  |
| Baseline | 21 | 3.9 (1.3) |  |  | 16 | 4.4 (1.9) |  |  | -0.5 [-1.6, 0.7] |
| 4 weeks | 20 | 3.4 (1.3) |  |  | 13 | 4.3 (2.0) |  |  | -0.8 [-2.2, 0.5] |
| 8 weeks | 19 | 3.0 (1.2) |  |  | 14 | 3.6 (1.6) |  |  | -0.6 [-1.6, 0.4] |
| Within arm change baseline vs. 8 weeks | 18 |  | -0.8 [-1.2, -0.3] | .001 | 13 |  | -0.4 [-0.8, 0.14] | .15 |  |

^a^ Mean value for within arm change between baseline and week 8.

^b^ Mean value for between-arm difference at baseline, week 4 and 8.
