# Supplementary material for: A Sustainable Lifestyle Intervention Among Office Workers: Cluster Randomized Pilot and Feasibility Study
Source: JMIR Form Res. 2026 May 7;10:e82061. doi: 10.2196/82061 (PMC13152203; doi:10.2196/82061)
Supplement: Multimedia Appendix 4 [file formative-v10-e82061-s004.docx]

**Multimedia Appendix 4:** Dietary goals reported by the sustainable and healthy lifestyle arm, analyzed and described as categories, codes and the number of goals per category for the two arms.

| **Sustainable lifestyle goals** | |  |  |
| --- | --- | --- | --- |
| **Categories** | **Codes** | | **Number of goals per category** |
| Increase intake of nutritionally dense foods | Nutritionally dense | | 3 |
| Increase vegetarian meals | Vegetarian Plant-based | | 4 |
| Increase fruits and vegetables | 500 grams of fruits and vegetables  More fruits and vegetables | | 5 |
| Increase intake of organically produced foods | KRAV Organic food | | 7 |
| Decrease the intake of red meat | Reduce read meat Less read meat | | 3 |
| **Healthy lifestyle goals** |  | |  |
| **Categories** | **Codes** | | **Number of goals** |
| According to the plate model | Plate model  Dinnerplate | | 3 |
| Decrease the intake of red meat to 500gram per week | 500 grams  Reduce read meat | | 4 |
| Increase the intake of nutritionally dense foods | Nutritionally dense  Nutritional value  Whole grain  Fiber | | 8 |
| 500 grams of fruits and vegetables per day | 500 grams  More vegetables and fruits | | 3 |
